# Dr. Omar E. Newman

**Published:** 1913-11

**Authors:** 


Dr. Omar E. Newman, Western Reserve, 1878, died at his home in Penn Yan, October 7, aged 55. He was born in Ontario County, graduated from the Canandaigua Academy in 1873. After graduating in medicine he commenced practice in Canandaigua and later moved to Potter. For the last twenty-five years he was a resident of Penn Yan. He was a member of the State and (Yates) County medical Societies.

